# Advances in the Regulation of Inflammatory Mediators in Nitric Oxide Synthase: Implications for Disease Modulation and Therapeutic Approaches

**DOI:** 10.3390/ijms26031204

**Published:** 2025-01-30

**Authors:** Mi Eun Kim, Jun Sik Lee

**Affiliations:** Immunology Research Lab, BK21-Four Educational Research Group for Age-associated Disorder Control Technology, Department of Biological Science, Chosun University, Gwangju 61452, Republic of Korea; kimme0303@chosun.ac.kr

**Keywords:** nitric oxide synthase, inflammatory mediators, therapeutic modulation

## Abstract

Nitric oxide synthases (NOS) are crucial enzymes responsible for the production of nitric oxide (NO), a signaling molecule with essential roles in vascular regulation, immune defense, and neurotransmission. The three NOS isoforms, endothelial NOS (eNOS), neuronal NOS (nNOS), and inducible NOS (iNOS), are tightly regulated by inflammatory mediators and cellular signaling pathways. While physiological NO production is vital for maintaining homeostasis, dysregulated NOS activity contributes to the pathogenesis of numerous diseases, including cardiovascular disorders, neurodegenerative conditions, and cancer. Recent advances in understanding the molecular mechanisms of NOS regulation have unveiled novel therapeutic opportunities, including isoform-specific modulators, upstream pathways, and nanotechnology-enhanced delivery systems. This review highlights these advancements, offering insights into how targeting NOS and its regulatory network can enable precise and effective therapeutic strategies for managing inflammation-driven pathologies.

## 1. Introduction

**Figure 1:**
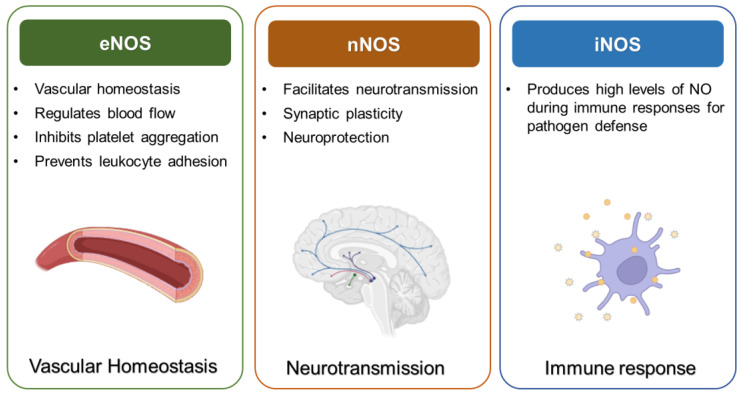
Simplified diagram of NOS isoforms and their roles. Roles of NOS isoforms: eNOS maintains vascular health, nNOS regulates neurotransmission, and iNOS drives immune responses. Dysregulation leads to diseases such as atherosclerosis (eNOS), neurodegeneration (nNOS), and chronic inflammation (iNOS), emphasizing the need for targeted therapies.

Nitric oxide (NO) is a versatile bioactive molecule integral to numerous physiological processes, including vascular tone regulation, neurotransmission, and immune defense [1]. NO is synthesized enzymatically by nitric oxide synthases (NOS), which exist in three distinct isoforms: endothelial NOS (eNOS), neuronal NOS (nNOS), and inducible NOS (iNOS). These isoforms, while sharing a common enzymatic function of converting L-arginine into L-citrulline and NO, exhibit unique expression patterns and regulatory mechanisms in different cellular contexts (Figure 1) [2,3].

The dysregulation of NOS has been implicated in the pathophysiology of various human diseases. eNOS dysfunction is a hallmark of cardiovascular disorders such as hypertension and atherosclerosis, while aberrant nNOS and iNOS activity contribute to neurodegenerative diseases and chronic inflammatory conditions, respectively. Inflammatory mediators, including cytokines, reactive oxygen species (ROS), and lipid-derived signals, significantly influence NOS activity, modulating NO production during both homeostasis and disease states [4].

NOS is currently a potential therapeutic target due to its important function in the pathophysiology of disease. Preclinical and clinical research have demonstrated the promise of approaches that modulate NOS activity, either by promoting protective eNOS function or by suppressing harmful iNOS activity. Additionally, novel possibilities for therapeutic intervention are presented by the interaction between NOS and inflammatory signaling pathways [4,5].

This review provides an inclusive analysis of recent advancements in understanding the regulatory mechanisms of nitric oxide synthases (NOS) by inflammatory mediators. It investigates the molecular mechanisms underlying these interactions and how they affect the progression of disease, as well as novel therapeutic approaches that target this regulatory network which have been developed.

## 2. Nitric Oxide Synthase and Inflammation

Nitric oxide synthases (NOS) are key enzymes responsible for the production of nitric oxide (NO), a signaling molecule with diverse roles in physiological and pathological processes. Among the three isoforms, endothelial NOS (eNOS), neuronal NOS (nNOS), and inducible NOS (iNOS), each has different regulations and performs particular functions. Their activities are finely tuned by inflammatory mediators, which modulate NO production under both normal and diseased states. Understanding whether inflammation influences NOS is essential to understanding its dual function in triggering disease and protecting homeostasis (Table 1) [6].

### 2.1. Types and Functions of NOS

The three isoforms of NOS differ in their tissue distribution, regulatory mechanisms, and contributions to inflammation and disease. eNOS is predominantly expressed in vascular endothelial cells and plays a central role in maintaining vascular integrity by regulating blood flow, inhibiting platelet aggregation, and preventing leukocyte adhesion [3,7]. Dysregulation of eNOS, particularly its uncoupling due to oxidative stress, results in reduced NO bioavailability and contributes to endothelial dysfunction, a key feature of cardiovascular diseases such as hypertension and atherosclerosis. In contrast, nNOS is primarily found in neurons, where it governs synaptic plasticity and neurotransmission [8]. Dysregulated nNOS activity is implicated in neurodegenerative disorders, where excessive NO production leads to excitotoxicity and neuronal damage. Unlike the constitutively expressed eNOS and nNOS, iNOS is induced in response to pro-inflammatory stimuli such as cytokines and microbial infections [4,9]. While iNOS-derived NO serves as an important defense mechanism against pathogens, its overproduction during chronic inflammation can result in tissue damage, as seen in autoimmune diseases like rheumatoid arthritis and inflammatory bowel disease [10].

### 2.2. Mechanisms of Inflammatory Regulation of NOS

Nitric oxide synthases (NOS) are tightly regulated by inflammatory processes that influence nitric oxide (NO) production through transcriptional mechanisms, post-translational modifications, and the availability of essential cofactors. These regulatory pathways ensure the balance between physiological NO production, critical for immune defense, vascular homeostasis, and tissue repair, and its pathological overproduction, which contributes to chronic diseases [8,11]. At the transcriptional level, the expression of inducible NOS (iNOS) is stimulated by pro-inflammatory cytokines such as TNF-α and IL-1β, which activate nuclear factor kappa B (NF-κB), a transcription factor that binds to the iNOS promoter in immune cells like macrophages. This activation drives robust NO production essential for microbial killing and immune signaling; however, prolonged NF-κB activation leads to excessive NO levels, resulting in tissue damage, oxidative stress, and chronic inflammation [12,13]. In conditions such as sepsis, persistent iNOS upregulation causes systemic vasodilation and organ dysfunction, while in rheumatoid arthritis, it exacerbates cartilage degradation and synovial inflammation [14]. Conversely, anti-inflammatory cytokines such as IL-10 counteract this process by inhibiting NF-κB, thereby suppressing iNOS transcription, reducing oxidative stress, and protecting tissue integrity during inflammation resolution [15].

In addition to transcriptional regulation, post-translational modifications play a critical role in dynamically modulating NOS activity, particularly endothelial NOS (eNOS), to ensure vascular homeostasis. Phosphorylation of eNOS by protein kinase (Akt) enhances its catalytic efficiency, promoting NO production necessary for vasodilation, inhibition of platelet aggregation, and prevention of leukocyte adhesion [1]. However, under inflammatory conditions, oxidative stress leads to eNOS uncoupling, a state where endothelial NOS (eNOS) produces superoxide (O_2_^−^) instead of nitric oxide (NO). This dysfunction occurs due to a deficiency in tetrahydrobiopterin (BH4), an essential cofactor for eNOS activity. Elevated levels of reactive oxygen species (ROS) oxidize BH4 into its inactive form, dihydrobiopterin (BH2). This shift in cofactor availability drives eNOS activity toward superoxide generation, exacerbating endothelial dysfunction, oxidative damage, and vascular inflammation. This cycle is a hallmark of cardiovascular diseases such as hypertension, atherosclerosis, and diabetes [16]. Further complicating eNOS regulation, S-nitrosylation and acetylation also influence its activity; while S-nitrosylation modulates protein interactions and NO signaling under normal conditions, excessive S-nitrosylation during inflammation disrupts eNOS function. Similarly, acetylation stabilizes eNOS, whereas deacetylation by SIRT1 enhances its activity under stress, with dysregulation of this balance impairing vascular responses [17].

The availability of cofactors such as BH4, flavin adenine dinucleotide (FAD), and nicotinamide adenine dinucleotide phosphate (NADPH) is equally critical for proper NOS function. Inflammatory oxidative stress depletes these cofactors, particularly BH4, further promoting eNOS uncoupling and reducing NO bioavailability [18]. Restorative strategies, including BH4 supplementation and antioxidants like ascorbic acid, have shown promise in experimental models, aiming to restore eNOS coupling, mitigate oxidative damage, and improve vascular function. Together, these multi-level regulatory mechanisms reveal the complexity of NOS modulation during inflammation. While these processes ensure precise NO control under physiological conditions, their dysregulation underlies a spectrum of acute and chronic pathologies, from sepsis to atherosclerosis and diabetes. Understanding these mechanisms provides a foundation for developing targeted therapeutic interventions to modulate NOS activity effectively in specific inflammatory contexts [19].

### 2.3. Dual Role of NOS in Inflammation

NOS play a dual role in inflammation, acting as both protectors and contributors to pathological processes, depending on the context and the isoform involved. This duality reflects the complex regulatory functions of NOS, which mediate protective responses during acute inflammation while driving tissue damage in chronic inflammatory states. Understanding this dual role is essential for designing targeted therapeutic interventions. In the vascular system, eNOS-derived NO is a key mediator of vascular homeostasis during acute inflammation. NO promotes vasodilation by activating soluble guanylate cyclase in vascular smooth muscle cells, improving blood flow and oxygen delivery to inflamed tissues [7]. It also reduces the expression of adhesion molecules such as VCAM-1 and ICAM-1 on endothelial cells, preventing the adhesion and infiltration of inflammatory leukocytes [5]. These effects limit endothelial damage and maintain vascular integrity, which are crucial for resolving inflammation and preventing excessive immune activation. Furthermore, eNOS-derived NO inhibits platelet aggregation, reducing the risk of thrombotic events that can exacerbate inflammatory injury. This protective role of eNOS is particularly evident in acute conditions, such as ischemia-reperfusion injury, where NO production mitigates oxidative stress and tissue damage [20]. nNOS contributes to neuroprotection during acute neuroinflammatory events by regulating cerebral blood flow and neuronal signaling. NO produced by nNOS induces vasodilation in cerebral vessels, ensuring adequate perfusion and oxygenation to neural tissues under inflammatory stress [21,22]. This protective mechanism minimizes ischemic injury and prevents the progression of neuroinflammation into chronic neurodegenerative conditions. Additionally, nNOS-derived NO modulates neurotransmitter release and synaptic activity, supporting neural recovery and maintaining homeostasis in the central nervous system (CNS) [7].

In contrast, iNOS is often overactivated during chronic inflammation, leading to excessive NO production and pathological consequences. While iNOS plays an essential role in host defense by producing large amounts of NO to combat pathogens, its persistent activity generates reactive nitrogen species that exacerbate oxidative damage. NO produced by iNOS reacts with O_2_^−^ to form peroxynitrite (ONOO^−^), a highly reactive molecule that damages lipids, proteins, and DNA. Lipid peroxidation compromises cell membranes, while protein nitration disrupts enzymatic functions and signaling pathways, collectively driving cellular dysfunction and apoptosis [23,24,25].

The detrimental effects of iNOS overactivation are evident in chronic inflammatory diseases. In atherosclerosis, iNOS-derived NO contributes to endothelial dysfunction and plaque formation by promoting oxidative stress and inflammatory cell recruitment to the vascular wall. Similarly, in rheumatoid arthritis, excessive NO levels exacerbate cartilage destruction and synovial inflammation, perpetuating joint damage and pain. In neurodegenerative diseases such as Alzheimer’s and Parkinson’s, iNOS overactivation amplifies microglial activation and inflammatory signaling, resulting in neuronal loss and cognitive decline [26,27,28,29]. The dual roles of NOS underscore the importance of tightly regulating its activity to maintain a balance between its protective and pathological functions. Enhancing eNOS and nNOS activity to preserve their beneficial effects while selectively inhibiting iNOS in chronic inflammatory conditions presents a promising therapeutic strategy. Targeted modulation of NOS activity could prevent tissue damage associated with chronic inflammation while maintaining the physiological benefits of NO during acute immune responses. Continued research into the mechanisms governing NOS regulation will be crucial for developing effective therapies that address the dual nature of its roles in inflammation.

## 3. Key Inflammatory Mediators in NOS Regulation

The regulation of NOS by inflammatory mediators plays a central role in controlling NO production during both homeostasis and disease. These mediators, which include cytokines, ROS, lipid-derived signals, and microRNAs (miRNAs), influence NOS activity at multiple levels, from transcription to enzymatic function. By modulating NOS activity, these inflammatory mediators contribute to the balance between protective NO signaling and pathological NO overproduction, highlighting their importance in health and disease [8].

### 3.1. Cytokines

Cytokines are critical regulators of NOS activity and can either amplify or suppress NO production depending on their inflammatory profiles. Pro-inflammatory cytokines such as tumor necrosis factor-alpha (TNF-α) and interleukin-1 beta (IL-1β) are potent inducers of inducible NOS (iNOS). TNF-α activates the transcription factor nuclear factor kappa B (NF-κB), which binds to the promoter region of the iNOS gene, initiating its transcription. This mechanism is further enhanced by IL-1β, which works synergistically with TNF-α to increase iNOS expression, particularly in macrophages during acute inflammation. While this response is crucial for host defense by generating NO to eliminate pathogens, chronic activation of these cytokines leads to excessive NO production, resulting in tissue damage and contributing to inflammatory diseases such as rheumatoid arthritis and inflammatory bowel disease [13,30,31]. In contrast, anti-inflammatory cytokines such as interleukin-10 (IL-10) and transforming growth factor-beta (TGF-β) act as natural brakes on NOS activity. IL-10 inhibits NF-κB activation, thereby suppressing iNOS transcription and reducing NO levels during prolonged inflammation. Similarly, TGF-β interferes with inflammatory signaling pathways, downregulating iNOS expression and mitigating NO-mediated oxidative stress. These cytokines protect tissues from excessive inflammation, illustrating the dual role of cytokine-mediated NOS regulation in balancing immune responses [32].

### 3.2. Lipid Mediators

Lipid-derived mediators, including prostaglandins and leukotrienes, play essential roles in regulating NOS activity during inflammation, exerting both protective and pathological effects depending on the inflammatory context. These bioactive lipids modulate NOS expression and function through intricate signaling pathways, highlighting their importance in acute and chronic inflammatory responses.

Prostaglandin E2 (PGE2), produced by the enzymatic action of cyclooxygenase-2 (COX-2), influences NOS activity by binding to its specific receptors, EP2 and EP4. These G-protein-coupled receptors activate the cyclic adenosine monophosphate–protein kinase A (cAMP-PKA) signaling pathway, which enhances the expression of iNOS in macrophages, leading to increase NO production. This mechanism is particularly important during acute inflammation, where elevated NO levels support pathogen clearance and immune cell recruitment. However, prolonged PGE2 signaling is associated with pathological outcomes, particularly in cancer. Sustained iNOS activation by PGE2 promotes angiogenesis through the upregulation of vascular endothelial growth factor (VEGF), facilitating tumor growth and metastasis. Additionally, chronic NO production in the tumor microenvironment suppresses anti-tumor immune responses, enabling immune evasion by cancer cells. These context-dependent effects of PGE2 demonstrate its dual role, acting as a protective mediator during acute inflammation but contributing to tumorigenesis under chronic conditions [33,34,35,36].

Similarly, leukotrienes, particularly leukotriene B4 (LTB4), significantly regulate NOS activity by promoting pro-inflammatory pathways. LTB4 induces the production of cytokines such as tumor necrosis factor-alpha (TNF-α) and interleukin-1 beta (IL-1β), which activate nuclear factor kappa B (NF-κB), a transcription factor that upregulates iNOS expression. This cascade enhances NO production, amplifying the inflammatory response during acute infections. While this is beneficial for immune activation and pathogen elimination, chronic elevations of LTB4 have been implicated in diseases such as asthma, rheumatoid arthritis, and inflammatory bowel disease. In rheumatoid arthritis, for example, LTB4-driven iNOS activation contributes to synovial inflammation, cartilage degradation, and joint destruction, perpetuating a cycle of chronic inflammation and tissue damage [37,38,39]. The interactions between lipid mediators and NOS reflect a delicate balance between their protective and pathological roles. During acute inflammation, the ability of PGE2 and LTB4 to enhance iNOS activity and NO production is critical for effective immune responses. However, in chronic conditions, sustained activation of these pathways results in excessive NO production, driving oxidative stress, tissue injury, and immune dysregulation. In cancer, persistent PGE2 signaling supports tumor progression by fostering an immunosuppressive environment, while elevated LTB4 levels in autoimmune diseases exacerbate inflammation and damage [40]. These findings underscore the need for context-specific therapeutic approaches targeting lipid mediator-NOS interactions. Selective COX-2 inhibitors, such as celecoxib, have shown promise in reducing PGE2-mediated iNOS activation, thereby mitigating inflammation and slowing tumor progression. Similarly, leukotriene receptor antagonists like montelukast effectively suppress LTB4-driven inflammation in conditions such as asthma and rheumatoid arthritis. These targeted strategies illustrate the potential for modulating lipid mediator-NOS pathways to treat chronic inflammatory and pathological conditions while preserving their beneficial effects during acute immune responses [41,42,43,44].

### 3.3. MicroRNAs (miRNAs)

MicroRNAs (miRNAs) have emerged as pivotal regulators of NOS activity, modulating gene expression at the post-transcriptional level to maintain a delicate balance between protective and pathological inflammatory responses. These small, non-coding RNAs influence the stability and translation of target mRNAs, providing a precise regulatory mechanism for NOS expression that is critical in both acute and chronic inflammation. Among the miRNAs involved in NOS regulation, miR-155 plays a prominent role in enhancing iNOS expression during inflammation. By stabilizing iNOS mRNA, miR-155 promotes increased NO production, a response particularly evident in sepsis and autoimmune diseases. Elevated levels of miR-155 are observed in immune cells exposed to pro-inflammatory stimuli such as lipopolysaccharides (LPS) and cytokines like TNF-α and IL-1β. While this mechanism supports pathogen clearance and immune activation, excessive NO production driven by miR-155 exacerbates nitrosative and oxidative stress, amplifying tissue damage and inflammatory responses. This has been particularly noted in conditions like rheumatoid arthritis, inflammatory bowel disease, and systemic lupus erythematosus, where the pathological effects of miR-155-mediated NO production are prominent. These findings underscore the therapeutic potential of targeting miR-155 to mitigate its role in excessive NO production and inflammatory damage [45,46,47].

Conversely, miR-146a acts as a counter-regulatory molecule, dampening inflammation by targeting key components of the NF-κB signaling pathway, such as interleukin-1 receptor-associated kinase 1 (IRAK1) and TNF receptor-associated factor 6 (TRAF6). By suppressing NF-κB activity, miR-146a reduces iNOS transcription and limits NO production, thereby protecting tissues from inflammation-induced damage. This anti-inflammatory role is particularly significant in the resolution phase of inflammation, where miR-146a aids in restoring tissue homeostasis and preventing chronic inflammatory conditions. Elevated levels of miR-146a have been linked to improved outcomes in sepsis and neuroinflammatory diseases, suggesting its therapeutic utility in conditions characterized by excessive or unresolved inflammation [48,49,50]. In addition to miR-155 and miR-146a, other miRNAs such as miR-21 and miR-27b contribute to the fine-tuning of NOS activity. miR-21 has been shown to suppress iNOS expression by targeting upstream pro-inflammatory signaling pathways, while miR-27b modulates both iNOS and eNOS expression depending on the cellular and inflammatory context. These additional layers of regulation highlight the complexity of miRNA-mediated NOS control and its significance in maintaining the balance between immune activation and resolution [51,52,53].

## 4. Role of NOS in Disease Pathogenesis

NOS play a central role in cardiovascular health, with their dysregulation contributing to various vascular and cardiac pathologies. eNOS is particularly critical in maintaining vascular homeostasis by producing NO, which promotes vasodilation, inhibits platelet aggregation, and prevents leukocyte adhesion to the endothelium. Through these actions, eNOS-derived NO ensures proper blood flow, reduces inflammation, and prevents thrombosis. However, in pathological conditions such as hypertension, diabetes, and atherosclerosis, increased oxidative stress depletes tetrahydrobiopterin (BH4), a key cofactor required for eNOS function. The loss of BH4 leads to eNOS uncoupling, a phenomenon in which eNOS produces O_2_^−^ instead of NO [54]. This shift not only reduces NO bioavailability but also exacerbates oxidative stress, promoting endothelial dysfunction and vascular inflammation. Endothelial dysfunction is a hallmark of atherosclerosis, where oxidative stress and inflammation initiate and drive the formation of atherosclerotic plaques. The impaired production of NO compromises vasodilation and promotes the adhesion of inflammatory cells to the endothelium, further exacerbating vascular damage. Strategies aimed at restoring eNOS activity have shown significant promise. For example, supplementation with BH4 or the use of antioxidants such as ascorbic acid has been shown to recouple eNOS, increasing NO production and mitigating oxidative damage. Additionally, pharmacological interventions like statins improve endothelial function by upregulating eNOS expression and enhancing its activity, providing cardiovascular protection beyond their lipid-lowering effects. These approaches underscore the therapeutic potential of targeting eNOS dysfunction in cardiovascular diseases [5,7,54]. In contrast to eNOS, inducible NOS (iNOS) plays a dual role in cardiac diseases, such as myocardial infarction and heart failure. During myocardial infarction, iNOS expression is upregulated in response to inflammatory signals, including cytokines such as TNF-α and IL-1β [55]. While moderate NO levels produced by iNOS can protect myocardial tissue by enhancing blood flow and reducing ischemia-reperfusion injury, excessive NO production leads to the formation of reactive nitrogen species, such as peroxynitrite. This nitrosative stress damages lipids, proteins, and DNA, impairing cardiac function and exacerbating tissue damage. In heart failure, persistent iNOS activation contributes to adverse cardiac remodeling, characterized by fibrosis, hypertrophy, and reduced contractility [27,56]. Given the dual role of iNOS, therapeutic strategies have focused on selectively inhibiting its pathological effects while preserving its protective functions. Selective iNOS inhibitors, such as 1400 W, have demonstrated potential in reducing nitrosative stress and improving cardiac function in preclinical models. These inhibitors target excessive NO production without interfering with baseline levels necessary for immune and repair processes, highlighting their potential as therapeutic agents for managing cardiac diseases such as MI and chronic heart failure [27,57]. The interplay between eNOS and iNOS in cardiovascular health accentuates the complexity of NOS regulation and its impact on disease progression. Developing targeted therapies to reduce vascular and cardiac damage while maintaining physiological NO activities requires an understanding of the complex roles of various isoforms (Table 2).

### 4.1. Neurological Disorders

NOS play a central role in neurological health and are key contributors to the pathogenesis of numerous neurodegenerative diseases and acute neurological injuries. The dysregulation of NOS activity disrupts neuronal function and exacerbates neuroinflammation, underscoring its dual roles in both protective and pathological processes within the CNS.

Neuronal NOS (nNOS) is essential for maintaining synaptic plasticity, neurotransmission, and overall neuronal health under physiological conditions. By producing moderate levels of NO, nNOS facilitates critical processes such as memory formation, long-term potentiation, and cerebral blood flow regulation. However, during excitotoxic events caused by ischemia, traumatic brain injury, or prolonged glutamate receptor activation, nNOS becomes overactivated, leading to excessive NO production. This excess NO reacts with ROS, such as superoxide, to form peroxynitrite, a highly reactive nitrogen species that induces oxidative and nitrosative stress. Peroxynitrite disrupts mitochondrial function, damages cellular components, and triggers neuronal apoptosis. This mechanism is strongly implicated in neurodegenerative diseases such as Alzheimer’s and Parkinson’s. In Alzheimer’s disease, nNOS overactivation exacerbates amyloid-beta-induced oxidative stress, promoting synaptic dysfunction and neuronal death, while in Parkinson’s disease, elevated nNOS activity contributes to dopaminergic neuronal degeneration through mitochondrial damage and neuroinflammation [58,59]. To address nNOS overactivation, selective inhibitors such as 7-nitroindazole (7-NI) have been explored as therapeutic agents. These inhibitors reduce excitotoxic damage by limiting excessive NO production without affecting the physiological functions of NO. Preclinical studies have demonstrated that 7-NI effectively mitigates oxidative stress, preserves mitochondrial integrity, and improves cognitive and motor outcomes in animal models of Alzheimer’s and Parkinson’s diseases [58,60].

iNOS plays a contrasting role in neurological disorders by driving neuroinflammation. Unlike nNOS, which is constitutively expressed, iNOS is upregulated in glial cells, including microglia and astrocytes, in response to inflammatory stimuli such as cytokines (e.g., TNF-α and IL-1β), LPS, or damage-associated molecular patterns (DAMPs). The resulting overproduction of NO by iNOS contributes to a pro-inflammatory environment, amplifying oxidative damage and exacerbating neuronal injury. In multiple sclerosis (MS), iNOS-derived NO disrupts the blood–brain barrier and accelerates demyelination, exacerbating neurodegeneration. Similarly, in ischemic stroke, elevated iNOS activity in the peri-infarct region contributes to secondary neuronal loss through enhanced oxidative stress. In traumatic brain injury (TBI), prolonged iNOS activation perpetuates inflammation, delaying recovery and exacerbating long-term neurological deficits [61,62,63].

### 4.2. Cancer

NOS exhibit a complex and context-dependent role in cancer, contributing to both tumor progression and suppression. The effects of NOS activity are influenced by the tumor microenvironment, the NOS isoform involved, and the levels of NO produced. Understanding these dynamics is essential for designing targeted therapies that leverage or inhibit NOS activity to improve cancer outcomes. iNOS is frequently upregulated in the tumor microenvironment, driven by inflammatory cytokines such as IL-6 and TNF-α, as well as hypoxic conditions that activate transcription factors like hypoxia-inducible factor-1 alpha (HIF-1α). Elevated iNOS expression results in high NO levels, which promote tumor progression through multiple mechanisms. NO enhances angiogenesis by upregulating vascular endothelial growth factor (VEGF) signaling, providing tumors with the blood supply necessary for growth and metastasis [64]. Additionally, NO fosters tumor cell proliferation by activating pro-survival pathways such as PI3K/Akt and MAPK signaling, while suppressing anti-tumor immune responses by impairing the activity of cytotoxic T-cells and natural killer (NK) cells [65,66]. Elevated iNOS levels have been associated with poor prognosis in several cancers, including breast and colorectal cancers. In breast cancer, high iNOS expression correlates with increased tumor aggressiveness and resistance to therapy, while in colorectal cancer, it has been linked to enhanced metastatic potential. These findings highlight iNOS as a potential therapeutic target in oncology. Strategies to inhibit iNOS activity, such as small-molecule inhibitors or RNA-based therapies, are being explored to suppress angiogenesis, reduce tumor growth, and restore immune surveillance within the tumor microenvironment [67,68].

nNOS and eNOS also play dual roles in cancer, contributing to both tumor progression and suppression depending on the context and levels of NO produced. At low to moderate concentrations, NO derived from nNOS and eNOS supports tumor progression by promoting angiogenesis and enhancing tumor cell survival [67]. For example, eNOS-derived NO facilitates endothelial cell proliferation and migration, which are critical for the formation of new blood vessels. Similarly, nNOS-derived NO can activate signaling pathways that increase tumor cell motility and invasiveness [67,69]. However, at higher concentrations, NO can exert cytotoxic effects on tumor cells and enhance anti-tumor immunity. In some contexts, nNOS activity has been shown to improve the efficacy of immune checkpoint inhibitors by enhancing T-cell infiltration into tumors. This mechanism may be particularly relevant in immunogenic tumors, where nNOS-derived NO supports immune-mediated tumor suppression. Similarly, eNOS can contribute to anti-tumor effects by improving blood flow within tumors, enhancing the delivery of immune cells and therapeutic agents [68,70,71].

## 5. Therapeutic Approaches Targeting NOS

The central role of NOS in numerous physiological and pathological processes has made them attractive targets for therapeutic intervention. Strategies for modulating NOS activity include enhancing beneficial functions, inhibiting pathological overproduction of NO, targeting upstream regulatory pathways, and employing advanced delivery systems. Each approach aims to achieve a delicate balance between the protective and harmful effects of NOS activity, depending on the context and disease state. This section outlines current and emerging therapeutic approaches targeting NOS, focusing on their mechanisms, potential applications, and limitations.

### 5.1. NOS Modulators

Direct modulation of NOS activity is a foundational approach in the therapeutic regulation of NO production, with selective inhibitors or enhancers of specific NOS isoforms offering targeted strategies for managing a range of diseases. The goal of these therapies is to reduce the pathogenic effects of NO while balancing its physiological benefits, depending on the condition.

Aminoguanidine works by directly inhibiting iNOS activity and also reducing advanced glycation end-product (AGE) formation, which is particularly relevant in diabetic complications. Similarly, 1400 W has shown potent and selective inhibition of iNOS, providing therapeutic benefits in models of inflammatory bowel disease and rheumatoid arthritis [72]. These inhibitors not only reduce inflammatory damage but also help restore tissue integrity and function. Despite their promise, translating these agents to clinical use requires overcoming challenges such as ensuring isoform specificity to avoid unintended inhibition of endothelial NOS (eNOS) or neuronal NOS (nNOS), which are critical for vascular and neural health. Emerging drug delivery systems, such as nanoparticles and liposomal carriers, are being developed to enhance the specificity, stability, and bioavailability of iNOS inhibitors, minimizing off-target effects and improving therapeutic outcomes [71,73]. In contrast to the harmful overactivation of iNOS, insufficient activity of eNOS is a key contributor to vascular dysfunction in diseases such as hypertension, diabetes, and atherosclerosis. eNOS-derived NO is essential for maintaining vascular homeostasis, promoting vasodilation, inhibiting platelet aggregation, and preventing leukocyte adhesion. However, oxidative stress, commonly observed in these diseases, depletes tetrahydrobiopterin (BH4), a critical cofactor for eNOS, leading to eNOS uncoupling. This uncoupling results in the production of superoxide instead of NO, further exacerbating oxidative stress and vascular damage [5].

Therapeutic strategies aimed at enhancing eNOS activity focus on restoring its function and improving NO bioavailability. Supplementation with BH4 has been shown to recouple eNOS, restoring its ability to produce NO and improving endothelial function in preclinical and clinical studies. For instance, in hypertensive and diabetic patients, BH4 supplementation has demonstrated reductions in oxidative stress and improvements in vascular reactivity.

Diarylheptanoids, particularly curcumin, are promising compounds for modulating nitric oxide synthase (NOS) activity due to their dual effects on iNOS and eNOS. Curcumin downregulates iNOS expression by inhibiting NF-κB, reducing excessive NO production and mitigating inflammation-related tissue damage. Simultaneously, it enhances eNOS activity through the PI3K/Akt pathway, promoting vasodilation and improving vascular health, particularly in conditions like atherosclerosis and hypertension. Curcumin also modulates oxidative stress by scavenging reactive oxygen species (ROS), preventing eNOS uncoupling, and increasing tetrahydrobiopterin (BH4) availability, crucial for endothelial integrity. Additionally, curcumin’s therapeutic potential is amplified when combined with other NOS-targeted therapies or delivered through nanotechnology-based systems, which enhance its stability and specificity [74].

Pharmacological agents that indirectly enhance eNOS activity have also proven effective. Statins, widely prescribed for their lipid-lowering properties, upregulate eNOS expression and enhance its phosphorylation at activation sites, leading to increased NO production and improved endothelial function. Similarly, angiotensin-converting enzyme (ACE) inhibitors improve eNOS activity by reducing oxidative stress, lowering blood pressure, and enhancing vascular integrity. The combined use of these agents provides synergistic benefits, not only addressing vascular dysfunction but also reducing the risk of cardiovascular events in patients with atherosclerosis and hypertension [75,76,77].

The ability to modulate NOS activity presents significant therapeutic potential across a wide range of diseases. However, the dual roles of NO being protective at physiological levels but harmful when dysregulated necessitate a nuanced approach to NOS targeted therapies. Isoform-specific modulation remains a primary goal to maximize therapeutic benefits while minimizing off-target effects. Advances in personalized medicine, such as the use of biomarkers to tailor NOS targeted treatments, are expected to improve the precision and efficacy of these interventions. Additionally, innovative drug delivery technologies, including liposomal formulations and nanoparticle-based systems, offer promising solutions for enhancing the stability, specificity, and targeted delivery of NOS modulators. These approaches aim to achieve controlled release and targeted action, minimizing systemic side effects while optimizing therapeutic outcomes. Continued research into the molecular mechanisms of NOS regulation and the development of advanced delivery platforms will be essential for fully realizing the potential of NOS modulators in clinical practice.

### 5.2. Targeting Upstream Regulators of NOS

Modulating upstream signaling pathways that regulate NOS expression and activity provides an alternative therapeutic approach, enabling the control of NOS-mediated effects without directly targeting the enzyme. This strategy focuses on the molecular drivers of NOS induction, such as pro-inflammatory cytokines and transcription factors, which are pivotal in modulating NOS activity in both physiological and pathological contexts.

Pro-inflammatory cytokines, particularly tumor necrosis factor-alpha (TNF-α) and interleukin-1 beta (IL-1β), are key drivers of inducible NOS (iNOS) expression in inflammatory diseases. These cytokines activate signaling cascades that enhance the transcription of the iNOS gene, leading to excessive nitric oxide (NO) production, oxidative stress, and tissue damage. Targeting these cytokines with biologics has proven effective in controlling iNOS activity and mitigating inflammation. TNF-α inhibitors, such as infliximab, neutralize the cytokine’s activity by binding to TNF-α and preventing it from interacting with its receptors. This suppression reduces the downstream induction of iNOS, alleviating inflammation and oxidative stress in autoimmune diseases such as rheumatoid arthritis and Crohn’s disease. Similarly, anakinra, an interleukin-1 receptor antagonist, blocks IL-1β signaling, which plays a critical role in amplifying iNOS expression. Clinical studies have demonstrated that these biologics significantly improve symptoms and reduce inflammatory markers in patients with chronic inflammatory diseases, highlighting their therapeutic efficacy [78].

In addition to cytokines, transcription factors such as NF-κB represent critical upstream regulators of iNOS expression. NF-κB is activated in response to inflammatory stimuli and binds to the promoter region of the iNOS gene, initiating its transcription. This process is a key mechanism driving excessive NO production in conditions such as sepsis, inflammatory bowel disease, and arthritis. Pharmacological inhibitors of NF-κB have shown potential in preclinical models for suppressing iNOS induction and reducing inflammation. For example, parthenolide, a naturally occurring NF-κB inhibitor, blocks the nuclear translocation of NF-κB, preventing it from activating iNOS expression. In animal models of sepsis and colitis, parthenolide has demonstrated efficacy in reducing oxidative damage and improving survival rates. These findings underscore the therapeutic potential of targeting NF-κB to regulate NOS activity and alleviate inflammatory conditions without directly inhibiting the enzyme itself [30,79].

Targeting upstream regulators of NOS provides a strategic advantage by addressing the molecular drivers of NOS induction rather than the enzyme directly. This approach minimizes the risk of disrupting physiological NO production mediated by eNOS and neuronal NOS (nNOS), which are essential for vascular and neural health. However, challenges remain in achieving specificity and minimizing off-target effects, particularly when targeting transcription factors like NF-κB, which regulate a broad range of genes. Advances in precision medicine and drug delivery systems, such as selective inhibitors and monoclonal antibodies, are paving the way for more targeted and effective modulation of upstream NOS regulators. These therapies hold significant potential for treating a variety of inflammatory and autoimmune diseases, offering a pathway to control NOS activity while preserving its physiological benefits.

### 5.3. Nanotechnology-Based Delivery Systems

Advances in nanotechnology have revolutionized the delivery of NOS modulators, offering enhanced specificity, controlled release, and minimized systemic side effects. Nanoparticle-based delivery systems encapsulate NOS inhibitors or enhancers, allowing targeted delivery to affected tissues while reducing off-target effects. These systems have shown significant potential in preclinical studies, addressing challenges associated with traditional therapies and paving the way for more precise and effective treatments.

Nanotechnology-based delivery systems have also been employed to address conditions characterized by reduced NO availability, such as ischemic heart disease, peripheral artery disease, and chronic wounds. Nanoparticles carrying NO donors, such as S-nitrosothiols or nitrate-based compounds, enable controlled and sustained release of NO at therapeutic levels. This approach ensures localized NO delivery, improving its bioavailability and minimizing the risk of systemic hypotension or other side effects [80]. In ischemic heart disease, NO-releasing nanoparticles have demonstrated improved endothelial function, enhanced angiogenesis, and reduced ischemic injury in preclinical models. Similarly, in wound healing, NO-releasing silica nanoparticles accelerate tissue repair by promoting angiogenesis and reducing bacterial infection at the wound site. These findings highlight the versatility of nanoparticle-based NO delivery in addressing a wide range of pathologies [81].

Nanotechnology-based delivery systems offer several advantages over conventional therapies, including improved drug stability, enhanced bioavailability, and precise targeting. These systems also enable dose optimization, reducing the frequency of administration and improving patient compliance. Moreover, advancements in biodegradable and biocompatible materials have further enhanced the safety profile of nanoparticle-based therapies. As research progresses, integrating nanotechnology with other cutting-edge approaches, such as gene editing or RNA-based therapies, could further expand the therapeutic potential of NOS modulation. For example, nanoparticles encapsulating small siRNA targeting iNOS mRNA could provide a novel strategy for selectively silencing iNOS expression in inflammatory diseases. These innovations underscore the transformative potential of nanotechnology in improving the precision and efficacy of NOS-targeted therapies.

### 5.4. Personalized Approaches in NOS-Targeted Therapies

The development of effective and precise NOS-targeted therapies hinges on a thorough understanding of specific biomarkers, genetic variations, and the integration of personalized medicine strategies. Biomarkers play a critical role in guiding these therapies by enabling patient stratification and monitoring treatment efficacy. For instance, nitrotyrosine, a marker of peroxynitrite formation, reflects NOS dysregulation and oxidative stress, providing valuable insights into the inflammatory status in conditions such as atherosclerosis and rheumatoid arthritis. Similarly, asymmetric dimethylarginine (ADMA), an endogenous inhibitor of NOS, is elevated in metabolic syndrome and cardiovascular diseases, signaling reduced NO bioavailability [82,83].

Beyond biomarkers, genetic variations in NOS isoforms significantly influence individual susceptibility to diseases and therapeutic outcomes. Polymorphisms such as T-786C and G894T in the eNOS (NOS3) gene are associated with impaired NO production and an increased risk of cardiovascular conditions, including hypertension and coronary artery disease. Similarly, polymorphisms in the iNOS (NOS2) gene affect inflammatory responses and are linked to autoimmune disorders like asthma and Crohn’s disease. Recognizing these genetic differences is essential for designing personalized therapeutic strategies, such as optimizing dosages or selecting specific NOS modulators tailored to a patient’s genetic profile [84].

The integration of NOS-targeted therapies with personalized medicine approaches presents a promising avenue for improving therapeutic outcomes. Combining NOS modulators with anti-inflammatory agents, such as NF-κB inhibitors or antioxidants, can synergistically reduce inflammation and oxidative stress. Furthermore, advancements in gene-editing technologies, such as CRISPR/Cas9, offer transformative potential by enabling the correction of pathogenic NOS3 variants or the upregulation of eNOS activity to restore vascular health. Nanotechnology-based delivery systems, including liposomes and nanoparticles, further enhance the precision of these therapies by enabling localized and targeted delivery of NOS modulators. This approach minimizes systemic side effects while improving therapeutic efficacy. By leveraging biomarkers, genetic insights, and advanced therapeutic platforms, personalized approaches in NOS-targeted therapies underscore the importance of tailoring treatments to individual patient needs. These strategies not only enhance the precision of interventions but also hold transformative potential in managing diseases characterized by NOS dysregulation, paving the way for more effective and patient-centered care.

## 6. Discussion

The regulation of nitric oxide synthases (NOS) by inflammatory mediators represents a critical intersection of physiology and pathology, with implications spanning cardiovascular health, neurodegeneration, immune defense, and cancer. This review consolidates recent advancements in understanding the mechanisms of NOS regulation and highlights emerging therapeutic strategies.

A key takeaway is the dual role of NOS, where its isoforms exhibit both protective and pathological effects depending on the context. Endothelial NOS (eNOS) plays a central role in vascular health by promoting vasodilation and preventing thrombosis, but its dysfunction due to oxidative stress or cofactor depletion exacerbates endothelial damage, contributing to diseases like atherosclerosis and diabetes. Similarly, neuronal NOS (nNOS) supports synaptic plasticity and neurotransmission but, when overactivated, contributes to excitotoxicity and neurodegeneration. Inducible NOS (iNOS), while essential for immune defense, becomes pathological in chronic inflammation, driving tissue damage and perpetuating conditions such as rheumatoid arthritis and inflammatory bowel disease. These observations underscore the necessity of isoform-specific approaches in therapeutic development.

Advances in therapeutic strategies targeting NOS demonstrate significant promise. Direct modulation of NOS activity through isoform-specific inhibitors or enhancers, such as iNOS inhibitors and eNOS activators, has shown efficacy in preclinical models of inflammation, cardiovascular diseases, and neurodegeneration. Additionally, targeting upstream regulators, including inflammatory cytokines like TNF-α and IL-1β or transcription factors like NF-κB, offers an alternative means to control NOS activity without direct enzymatic inhibition. These upstream interventions, such as TNF inhibitors and NF-κB blockers, have shown potential in reducing pathological NO production and mitigating inflammation in autoimmune diseases and sepsis [12,13,14,15].

Innovations in nanotechnology-based delivery systems further enhance the precision of NOS-targeted therapies. Encapsulation of NOS modulators within nanoparticles has improved tissue-specific delivery, bioavailability, and control over drug release. For instance, NO-releasing nanoparticles have demonstrated therapeutic benefits in ischemic heart disease and wound healing, while liposomal formulations of iNOS inhibitors have shown efficacy in reducing inflammation in arthritis models. Such technologies minimize systemic side effects and maximize therapeutic outcomes, heralding a new era in NOS-targeted treatments [71,79,80].

Despite these advances, challenges remain. Achieving isoform specificity, minimizing off-target effects, and optimizing delivery systems are critical hurdles in translating these therapies into clinical practice. Moreover, the complexity of NOS regulation, involving transcriptional, post-transcriptional, and post-translational mechanisms, necessitates a nuanced understanding of its biology for effective intervention. Future research should focus on integrating multimodal approaches, combining pharmacological, nanotechnological, and genetic strategies to address NOS dysregulation comprehensively. Personalized medicine approaches, leveraging biomarkers to tailor NOS-targeted therapies, also hold promise for enhancing treatment precision and efficacy.

## 7. Conclusions

NOS serve as pivotal regulators of cellular and systemic processes, with their dysregulation linked to the progression of various chronic and acute diseases. Advances in understanding the regulatory mechanisms of NOS, particularly in inflammatory contexts, have revealed its dual roles in maintaining physiological homeostasis and driving pathological processes. Therapeutic strategies targeting NOS have progressed from direct modulators of enzymatic activity to sophisticated approaches that include NOS modulators, upstream regulators, and nanotechnology-enhanced delivery systems. These advancements underscore the importance of precision medicine in addressing the complex interplay between NOS isoforms, inflammatory mediators, and disease mechanisms. Future research should focus on refining isoform-specific therapies, integrating multimodal approaches, and optimizing delivery systems to maximize therapeutic efficacy while minimizing off-target effects. By leveraging these innovations, the clinical potential of NOS-targeted therapies can be fully realized, offering transformative solutions for diseases where inflammation and oxidative stress play central roles.

## Figures and Tables

**Table 1:** Inflammatory regulation of NOS.

Regulatory Mechanism	Effect	Examples
Cytokines	Pro-inflammatory cytokines (e.g., TNF-α, IL-1β) induce iNOS; anti-inflammatory cytokines (e.g., IL-10) suppress iNOS	TNF-α promotes NF-κB activation; IL-10 inhibits NF-κB and reduces NO production
Lipid Mediators	Prostaglandins (e.g., PGE2) and leukotrienes (e.g., LTB4) modulate NOS through receptor-mediated signaling pathways	PGE2 enhances iNOS expression; LTB4 promotes inflammation in asthma and arthritis
MicroRNAs	miRNAs like miR-155 enhance iNOS, while miR-146a suppresses NF-κB signaling, reducing iNOS expression	miR-155 linked to autoimmune diseases; miR-146a protective in neuroinflammation

**Table 2:** Disease-specific applications.

Disease	Pathological Role of NOS	Therapeutic Strategy
Cardiovascular Diseases	eNOS dysfunction leads to reduced NO, endothelial damage, and vascular inflammation	BH4 supplementation, statins, and ACE inhibitors to restore NO bioavailability and vascular function
Neurodegenerative Disorders	Overactive nNOS and iNOS contribute to oxidative stress and neuroinflammation	Selective nNOS inhibitors (e.g., 7-NI), siRNA targeting iNOS to reduce nitrosative stress
Cancer	iNOS promotes angiogenesis and immune evasion; nNOS/eNOS have dual roles in tumor growth and immune responses	iNOS inhibitors to suppress tumor progression; leveraging nNOS/eNOS for enhancing immunotherapy
Inflammatory Diseases	iNOS overactivation exacerbates chronic inflammation and tissue damage	NF-κB inhibitors and RNA-based therapies to downregulate iNOS expression

## Data Availability

The data presented in this study are available on request from the corresponding author. The data are not publicly available due to privacy.

## Abbreviations

ADMA: asymmetric dimethylarginine, Akt: protein kinase, BH4: tetrahydrobiopterin, CNS: central nervous system, COX-2: cyclooxygenase-2, DAMPs: damage-associated molecular patterns, eNOS: endothelial nitric oxide synthase, HIF-1α: hypoxia-inducible factor-1 alpha, ICAM-1: intercellular adhesion molecule-1, IL: interleukin, iNOS: inducible nitric oxide synthase, LPS: lipopolysaccharides, MAPK: mitogen-activated protein kinase, miRNA: microRNA, NF-κB: nuclear factor kappa B, NK cells: natural killer cells, NO: nitric oxide, NOS: nitric oxide synthase, nNOS: neuronal nitric oxide synthase, PGE2: prostaglandin E2, PI3K: phosphoinositide 3-Kinase, ROS: reactive oxygen species, SIRT1: sirtuin 1, TNF-α: tumor necrosis factor-alpha, VCAM-1: vascular cell adhesion molecule-1, VEGF: vascular endothelial growth factor.
